# Cell Membrane-Coated Nanoparticles As an Emerging Antibacterial Vaccine Platform

**DOI:** 10.3390/vaccines3040814

**Published:** 2015-10-06

**Authors:** Pavimol Angsantikul, Soracha Thamphiwatana, Weiwei Gao, Liangfang Zhang

**Affiliations:** Department of NanoEngineering and Moores Cancer Center, University of California, La Jolla, San Diego, CA 92093, USA; E-Mails: pangsant@ucsd.edu (P.A.); sthamphi@ucsd.edu (S.T.); w5gao@ucsd.edu (W.G.)

**Keywords:** nanomedicine, nanovaccine, biomimetic nanoparticle, membrane coating, infectious disease

## Abstract

Nanoparticles have demonstrated unique advantages in enhancing immunotherapy potency and have drawn increasing interest in developing safe and effective vaccine formulations. Recent technological advancement has led to the discovery and development of cell membrane-coated nanoparticles, which combine the rich functionalities of cellular membranes and the engineering flexibility of synthetic nanomaterials. This new class of biomimetic nanoparticles has inspired novel vaccine design strategies with strong potential for modulating antibacterial immunity. This article will review recent progress on using cell membrane-coated nanoparticles for antibacterial vaccination. Specifically, two major development strategies will be discussed, namely (i) vaccination against virulence factors through bacterial toxin sequestration; and (ii) vaccination against pathogens through mimicking bacterial antigen presentation.

## 1. Introduction

Antibacterial vaccines are considered the most cost-effective intervention against bacterial infections. They have achieved remarkable success in controlling past epidemics worldwide [1]. In current practice, the use of antibacterial vaccines, including whole-cell bacteria, killed or inactivated bacteria, subunit vaccines made of bacterial proteins or polysaccharides, and inactivated bacterial toxins (toxoids), have drastically reduced global morbidity and mortality [1,2]. Despite such success, effective vaccines remain largely unavailable for the treatment and prevention of a number of serious bacterial infections, including those caused by pathogenic *Staphylococcus aureus, Helicobacter pylori*, *Shigella*, and *Escherichia coli* (*E. coli*) [3,4]. Meanwhile, currently available antibiotic regimens are increasingly threatened by the rapid emergence of bacterial drug resistance [5,6]. These challenges, together, have motivated the search for novel antibacterial vaccine strategies.

On the other hand, nanotechnology has been rapidly developed and successfully used in biomedical research. A large amount of effort has been directed toward leveraging nanoparticle technology for vaccine development [7,8]. These emerging nanoparticle-based vaccines exhibit unique properties that may enable them to outperform traditional vaccine formulations [9,10]. For instance, nanoparticles can concurrently carry antigens and immunostimulatory adjuvants to mimic natural microbes for eliciting superior immune responses [11]. Programmed release of these immune determinants further allows for coordinated immune activation, leading to effective vaccination efficacy [12,13,14]. Targeted nanoparticles can lead to enhanced accumulation in a specific organ, tissue, or cell type, which facilitates their uptake by antigen-presenting cells (APCs) to induce more effective T cell responses [15,16,17]. Furthermore, environment-responsive nanoparticles, when exposed to external stimuli, can change material structure and surface characteristics in a manner that favors site-specific deposition of vaccine payloads for immune activation [18,19,20]. More recently, a few nanoparticle systems with elegant intra-particle architectures have been developed that transform nanoparticles from simple cargo carriers to sophisticated antigen-presenting entities eliciting strong humoral and cytotoxic T lymphocyte responses [21,22,23].

**Figure 1:**
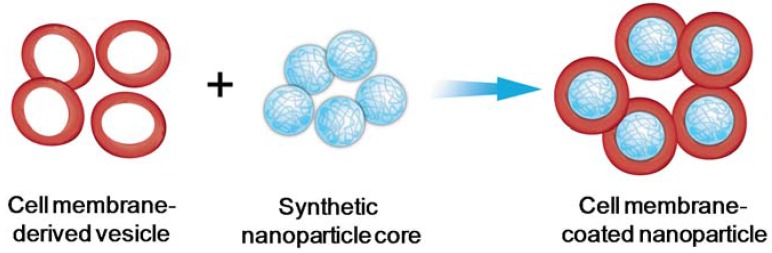
Schematic preparation of cell membrane-coated nanoparticles. Briefly, cell membrane-derived vesicles along with the associated membrane antigens are collected from source cells and then coated onto the surface of pre-formed synthetic nanoparticles.

Among various nanoparticle vaccine platforms, one emerging design is the use of natural cell membranes to coat synthetic nanoparticles [24,25,26]. In this design, intact cell membranes are collected from natural cells and then coated onto nanoparticle surface (Figure 1). The resulting cell membrane-coated nanoparticles preserve the highly tunable physicochemical properties of synthetic nanomaterials while inheriting complex cellular surface antigens and functions. The natural membranes also provide a bilayered medium ideal for transmembrane protein anchorage and prevent the loss of integrity and functionalities of the proteins during both formulation preparation and delivery processes. The separate preparation of cell membrane-derived vesicles and nanoparticle cores prior to fusing them together offers additional engineering flexibility toward making highly functional biomimetic nanoparticles.

The unique design of cell membrane-coated nanoparticles has unleashed promising potential of exploiting natural functionalities of the source cells for innovative applications, especially in treating bacterial infections. While this class of biomimetic nanoparticles was developed not long ago, it has already demonstrated strength as effective antibacterial vaccines. In this article, we will review recent progress made in using this class of nanoparticles as an emerging vaccine platform against bacterial infections. Two major development strategies will be highlighted, namely (i) vaccination against virulence factors through bacterial toxin sequestration, and (ii) vaccination against pathogens through mimicking bacterial antigen presentation. Overall, cell membrane-coated nanoparticles have emerged as a novel class of vaccine platform with promising potential for effective antibacterial immunization.

## 2. Vaccination Against Virulence Factors through Bacterial Toxin Sequestration

**Figure 2:**
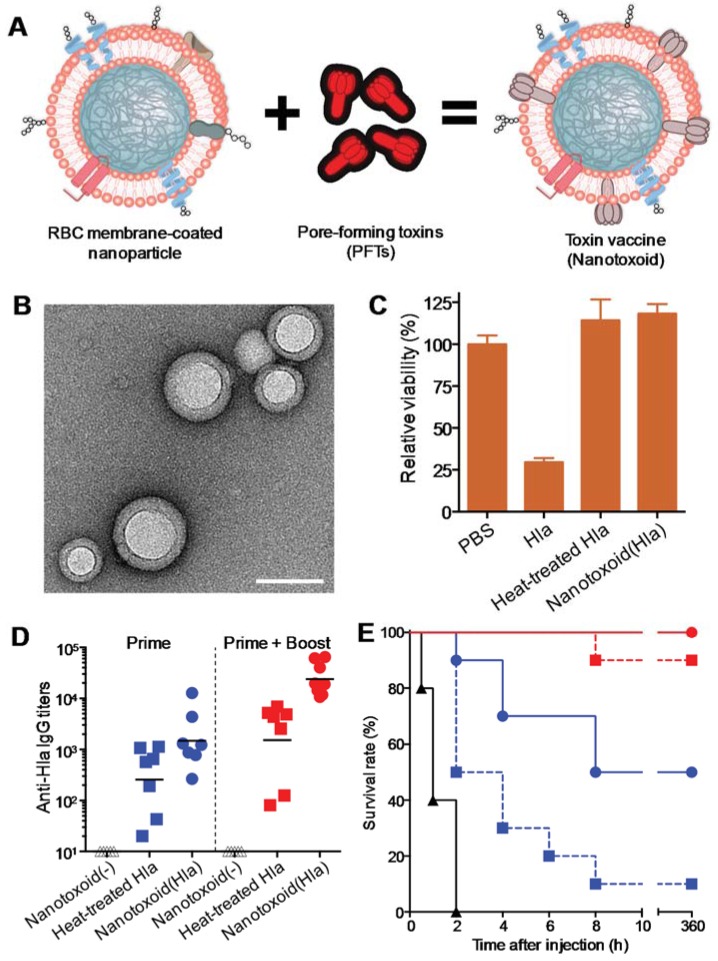
(**A**) Schematic preparation of nanoparticle-detained toxins, denoted “nanotoxoid”, consisting of substrate-supported RBC membranes into which pore-forming toxins (PFTs) can spontaneously incorporate; (**B**) A transmission electron microscopic (TEM) image of the particle vectors with uranyl-acetate staining (scale bar, 80 nm); (**C**) Toxicity of different Hlα formulations against DCs derived from mice. The cells were incubated for 48 h with Hlα, heat-treated Hlα (60 min) or nanotoxoid (Hlα) at 15 μg/mL Hlα concentration. Cellular viability was assessed using an MTT assay (*n* = 6); (**D**) Vaccination was conducted following two different schedules: a prime only on day 0 and a prime on day 0 plus two booster vaccinations on day 7 and day 14, respectively. Anti- Hlα IgG titers in the vaccinated mice were quantified on day 21 (*n* = 7); (**E**) Survival rates of mice over a 15-day period following intravenous injections of 120 μg/kg Hlα on day 21 via the tail vein (*n* = 10). The unvaccinated mice were used as a negative control and mice vaccinated with heat-treated Hlα served as positive controls. Reproduced with permission from Reference [26].

Following their initial development, cell membrane-coated nanoparticles were shown to sequester cytotoxic virulent factors including bacterial toxins. On nanoparticle interaction, intact, non-denatured toxins lose their motional freedom and are “detained” by the cell membrane-coated nanoparticles. These detained toxins are precluded from initiating their normal virulence mechanisms and can thus be safely delivered *in vivo* for effective immune processing (Figure 2A) [26]. Such toxin-detainment strategy adds a new dimension to nanoparticulate vaccines, which have previously focused on applying nanoparticles as passive carriers for antigens with weakened immunogenicity. The toxin-nanoparticle complex, denoted “nanotoxoid”, has profound implications in the preparation of toxoid vaccines, which can be applied for the treatment and prevention of various types of bacterial infections [27].

Bacterial toxins can alter the normal metabolism of host cells and have been identified as the primary causative factors in many infectious diseases [28]. The role of toxins in infections has prompted the development of toxoid vaccines [29,30,31], which are inactivated forms of toxins that can be administered to mount an anti-toxin immune response. Conventional toxoid preparation methods involve using heat or chemical treatment to denature protein toxins for neutralizing their virulence. Chemical- and heat-mediated detoxification processes are difficult to fine-tune, and they are known to disrupt a protein’s tertiary structure, causing altered antigenic presentation and compromised immunogenicity [32,33,34]. The shortfalls of the conventional toxoid preparation are evidenced in the decades-long effort in the development of α-hemolysin (Hlα) toxoid against *Staphylococcus aureus* infections, as early development of denaturation-based Hlα toxoid vaccines were associated with either residual toxicity or inadequate potency [35]. More recent efforts have focused on the development of non-toxic but structurally conserved toxin mutants using advanced biomolecular techniques. In particular, site-directed mutagenesis has been applied to produce toxin mutants with minimal antigenic alterations from the target toxins, thereby minimizing the tradeoff between safety and efficacy [36,37,38,39]. In nanoparticle-detainment strategy, particle carriers are applied to intercept toxins’ virulence mechanism, thereby enabling unaltered toxins to be administered for immune processing.

By using Hlα as a model toxin, we recently demonstrated successful toxin detainment with a red blood cell (RBC) membrane-coated nanoparticle platform, which consists of natural RBC membranes wrapped on sub-100 nm biodegradable poly(lactic-co-glycolic acid) (PLGA) polymeric cores (Figure 2B). The RBC membrane-coated nanoparticles are enclosed by a unilamellar biomembrane bilayer, which serves as a substrate for spontaneous toxin interactions. The membrane-targeting Hlα readily inserted into the RBC membranes and were sequestered by the stable particle structure. Each nanoparticle was found to adsorb dozens of toxin monomers, and toxin detoxification could be achieved in a facile and reliable manner by mixing the toxin with a sufficient number of nanoparticles [26]. Upon toxin absorption and detainment, the resulting Hlα-loaded nanotoxoid showed no observable toxicity. In contrast to the rapid and effective detoxification via the nanoparticle detainment approach, heat inactivation required at least 60 min of heating at 70 °C for toxin neutralization (Figure 2C). As detained toxins retain their protein structure, mice vaccinated with nanotoxoid(Hlα) generated significantly higher anti-Hlα immune responses as compared to those vaccinated with heat-denatured Hlα (Figure 2D). Most impressively, mice receiving three weekly doses nanotoxoid(Hlα) vaccine became completely immune to the toxin (Figure 2E). High doses of Hlα that can cause serious tissue damage in non-vaccinated mice did not inflict any observable effect in the vaccinated mice upon subcutaneous injections.

The biocompatible nature of RBC membranes and PLGA polymers allow the immune system to selectively process the toxin cargoes without provoking an immune response against the nanoparticle carrier. No anti-nanoparticle immune response was observed despite the high anti-toxin responses generated by the nanotoxoid. As a platform technique, the RBC membrane-coated nanoparticles also allow for the detainment of other membrane-active protein toxins. In an earlier work, we demonstrated successful neutralization of two other types of pore-forming toxins, an oligomerizing streptolysin-O from Streptococcus bacteria and a small peptide from bee venom [25]. Given the broad presence of membrane-targeting virulence factors in pathogenic microbes, such as *Escherichia coli*, *Helicobacter pylori*, *Clostridium perfringens*, and *Bacillus anthracis* [40], the biomimetic nanoparticle system offers a versatile approach for vaccine development against many infectious diseases. In addition to the membrane-coated exterior that serves to sequester pore-forming toxins, the nanoparticles possess other characteristic properties that enhance the immune processing of the toxin antigens. For instance, owing to the nanoparticles’ stability and small size, they are able to facilitate the antigen delivery to lymphatic organs such as the spleen and the lymph nodes [26]. The nanoparticle/toxin complexes also possess a particulate morphology that is more prone to cellular ingestion as compared to free proteins [41]. This property allows toxin antigens to be efficiently taken up and metabolized by antigen presenting cells for immune processing. Along with the antigenically preserved toxin antigens, these other factors likely contributed to the enhanced antibody responses.

The ability to neutralize toxins via the detainment strategy also highlights the intricate biomolecular machineries behind the virulence mechanisms of protein toxins [42]. For instance, pore-forming toxins, such as Hlα and streptolysin-O, require membrane interactions and oligomerizing actions with other toxin monomers for channel formation and cellular disruption. Nanoparticle detainment functions as a toxin decoy to absorb toxins and constrain toxins’ freedom, thereby diverting them away from targeted cellular substrates. It can be envisioned that this detainment concept may be extended to other non-pore-forming toxins that require interactions with specific substrates and receptors to take effect. For example, toxins that interact with membrane receptors (*i.e.*, neurotoxin) or cytosolic substrates (*i.e.*, Shiga toxin) can likewise be detained by nanoparticles to preclude their virulence activities and to facilitate their cellular digestion and immune processing. Toward future development, however, rigorous safety characterizations of particle-detained formulations are warranted as sequestered toxins can be potentially bioactive. Methods that help secure the toxin detainment, enhance particle stability, and accelerate particle cellular uptake are expected to benefit the overall vaccine system as they minimize the risks of premature toxin release. Given the synthetic flexibility of nanomaterials, numerous toxin association and immune modulation approaches are possible [43]. As antibiotic resistance poses a rising threat that is claiming millions of lives per year, the urgent need for emerging antimicrobial measures can benefit from creative engineering in nanotechnology. As the nanoparticle-mediated toxin detainment approach promises vaccine formulations with higher potency, we anticipate further development of the new nanotoxoid vaccines can improve the current management of infectious diseases. By promoting anti-virulence immunity against pathogenic factors of bacteria, the vaccination approach could reduce the occurrence of microbial infections without reliance on antibiotics.

## 3. Vaccination against Pathogens through Mimicking Bacterial Antigen Presentation

An exciting aspect of nanoparticles is their ability to mimic key aspects of bacterial pathogens to induce effective antibacterial immunity [44,45,46]. Synthetic nanoparticles can carry protein, peptide, and DNA antigens along with various immune-potentiating components, which together mimic bacterial composition for immune activation. In addition, nanoparticles can mimic bacteria in presenting multiple peptide epitopes in a repetitive pattern, which could improve the poor immunogenicity currently inherent in peptide subunit vaccines [47,48]. By mimicking bacteria, nanoparticles can also directly target APCs with both T and B cell epitopes, which induce both cellular and humoral immune responses essential for treatment of various bacterial infections [47,49].

Despite these attractive features, a major challenge in designing bacterium-mimicking nanoparticles is to balance the faithful replication of bacterial immune characteristics and the preservation of engineering flexibility and controllability for immune modulations [50,51]. To address this challenge, a unique bacterial membrane-coated nanoparticle system has been recently developed and tested as a new antibacterial vaccine platform [52]. Bacterial membranes are appealing vaccination materials as they contain a large number of immunogenic antigens with intrinsic adjuvant properties [53]. Bacterial membranes also exhibit various pathogen associated-molecular patterns that play a key role in stimulating innate immunity and promoting adaptive immune responses [54,55]. When bacterial membranes are coated onto the surfaces of synthetic nanoparticles, these particles are disguised as real pathogens. They preserve the complex biological characteristics of bacteria and present the natural antigens to the immune system. Meanwhile, the synthetic nanoparticle cores provide a wide range of tunable physicochemical properties such as particle size and shape for effective antigen presentation to the immune cells [56,57]. Therefore, the bacterial membrane-coated nanoparticles combine the merits of two distinct nanomaterials and are expected to generate strong antibacterial immune responses.

**Figure 3:**
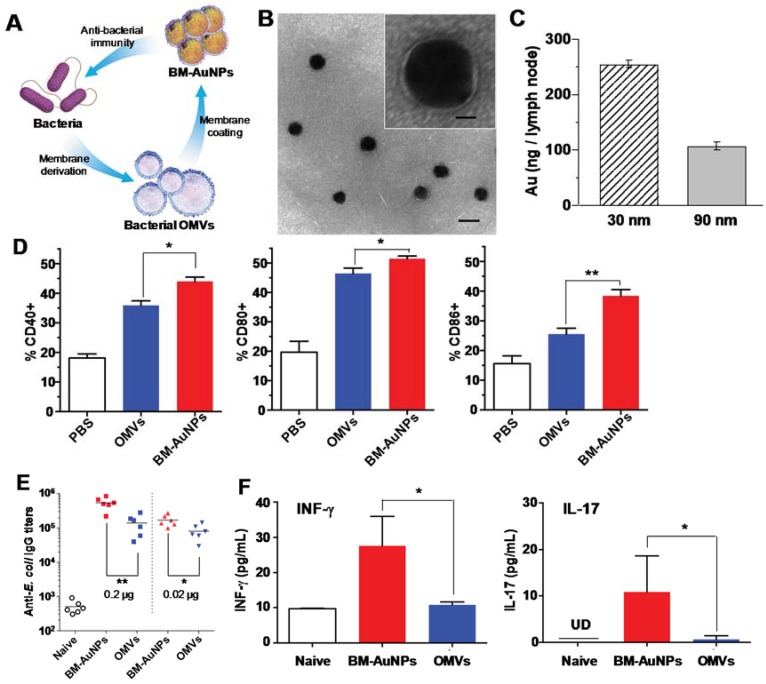
(**A**) A schematic illustration of modulating antibacterial immunity via bacterial membrane-coated nanoparticles. Briefly, bacterial membrane is collected from source bacteria in the form of secreted outer membrane vesicles (OMVs) and then coated onto citrate-stabilized gold nanoparticles (AuNPs) to form bacterial membrane-coated AuNPs (BM-AuNPs). When injected subcutaneously into mice, BM-AuNPs elicit bacterium-specific immunity against the source bacteria; (**B**) A representative TEM image showing the spherical core-shell structure of the BM-AuNPs negatively stained with uranyl acetate (scale bar, 50 nm). Inset: a zoomed-in view of a single BM-AuNP (scale bar, 10 nm); (**C**) Effect of BM-AuNP size on its lymph node transport. Mice were injected with 2.5 μg (50 μg/mL) BM-AuNPs of 30 nm and 90 nm, respectively, via the tail base. After 24 h, the lumbar and sacral lymph nodes were collected. Gold concentration was quantified by using inductively coupled plasma mass spectrometry (ICP*-*MS); (**D**) Quantification of the percentage of CD11c+ DCs based on flow cytometry analysis of surface maturation markers (CD40, CD80, and CD86) on CD11c+ DCs from lumbar and sacral lymph nodes of the mice (*n* = 5); (**E**) BM-AuNPs eliciting strong anti-*E. coli* IgG titres *in vivo* on day 21 (*n* = 6); (**F**) BM-AuNPs inducing pronounced bacterium-specific T cell activation *in vivo*. The mice (*n* = 10) were immunized with 0.2-μg antigen/dose BM-AuNPs or OMVs. On day 21, splenic cells were collected and stimulated with *E. coli* bacteria. After 72 h of co-culturing with the bacteria, the levels of IFN-γ and IL-17 in the medium were quantified using an ELISA. Reproduced with permission from Reference [52].

We recently chose *E. coli* bacteria as a model pathogen and harnessed their outer membranes through the collection of their secreted outer membrane vesicles (OMVs). Originating from bacterial outer membranes, OMVs share similar biochemical profiles with their parent cells [54,55]. They are known to generate potent protective immune responses against the source pathogens with particular success in treating *Neisseria meningitides* [58,59]. Furthermore, *Shigella* OMVs encapsulated in poly(anhydride) nanoparticles have shown enhanced mucosal protection compared to free OMVs in a mouse model [60]. However, to treat a wider range of pathogen infections, novel strategies to manipulate OMVs and modulate the subsequent immune responses are highly desirable. As illustrated in Figure 3A, the collected bacterial OMVs were coated onto the surfaces of small gold nanoparticles (AuNPs). The AuNPs were chosen because their size and shape could be precisely tailored to favor immunization applications [61,62]. In the study, we demonstrated successful coating of bacterial membranes onto small AuNPs with a diameter of 30 nm (Figure 3B). When subcutaneously injected into mice, the bacterial membrane-coated AuNPs (BM-AuNPs) traveled to adjacent draining lymph nodes in a particle size dependent manner. Specifically, we found that BM-AuNPs made with smaller AuNPs (30 nm in diameter) showed a significantly enhanced accumulation in the lymph nodes of mice as compared to larger BM-AuNPs made with 90 nm AuNPs (Figure 3C). We further compared the efficacy of BM-AuNPs and OMVs in eliciting DC maturation. In both groups, CD11c+ DCs isolated from lumbar and sacral lymph nodes displayed a shift from the immature to the mature phenotype through upregulation of the co-stimulatory molecules CD40, CD80, and CD86. Further quantification of CD11c+ DCs based on the histograms showed that the DC maturation level induced by BM-AuNPs was significantly higher than that induced by the OMVs alone (Figure 3D). We next assessed bacterium-specific B cell responses by examining the elicitation of *E. coli*-specific antibodies. Determination of antibody responses on day 21 showed that BM-AuNPs induced significantly higher *E. coli*-specific antibody titers in comparison to the OMVs (Figure 3E). We also examined the effects of BM-AuNP immunization on activating bacterium-specific T cell responses. The results showed that the levels of IFN-γ and IL-17 production were higher in mice immunized with either BM-AuNPs or OMVs compared to the naïve mice, indicating successful *E. coli*-specific T cell activation. The comparison also showed that mice immunized with BM-AuNPs produced significantly higher levels of IFN-γ and IL-17 than OMV-immunized mice, implying a higher efficacy of BM-AuNPs in activating T cells (Figure 3F). Collectively, these results indicate that coating natural bacterial membranes onto synthetic nanoparticles represent a promising approach to developing an antibacterial vaccine toward effective treatment of bacterial infection. 

The design of BM-AuNPs seeks synergy between bacterial membranes and synthetic nanoparticles to generate a new and effective antibacterial vaccine. Using bacterial OMVs as membrane materials, BM-AuNPs contain a large number of immunogenic antigens with intrinsic adjuvant properties. In addition, the faithful translocation of the entire bacterial membranes onto the nanoparticle surfaces preserves critical immune determinants, such as the pathogen associated-molecular patterns. As a result, the BM-AuNPs closely mimic antigen presentation by bacteria to the immune cells. Meanwhile, using uniformly distributed synthetic nanoparticles such as AuNPs as coating template allows for a wide range of controllability over the physicochemical properties of the vaccine formulation. In this study, the AuNP-templated membrane coating transformed OMVs from widely polydispersed vesicles into uniformly distributed ultrasmall nanoparticles and subsequently resulted in rapid DC activation *in vivo*. Moreover, the AuNP-templated coating led to strong association between the membranes and the cores, which likely reinforced the multivalent display of epitopes on the bacterial membranes. Such strengthened interactions between the antigens and the nanoparticle architecture have been attributed to the enhanced antibody and endogenous T cell responses in other nanoparticle-based vaccine platforms [21,22,26]. Overall, the bacterial membranes and the nanoparticle cores mutually benefit each other, synergistically generating enhanced antibacterial immune responses.

Using bacterial membranes to coat synthetic nanoparticles opens new opportunities for designing effective antibacterial vaccines. For example, secretion of extracellular vesicles (EVs) represents a conserved process found on both Gram-negative (e.g., OMVs) and Gram-positive bacteria (e.g., membrane-derived vesicles) [63]. Therefore, this reported technique can potentially be applied to various types of bacteria [64,65]. In addition, genetic engineering has been successful in developing EVs that express multiple mutants or exogenous antigens [66,67]. These engineered EVs can be also exploited as membrane materials for broadening immune protection. From a translational perspective, high yield and large-scale manufacturing processes of EVs are under rapid development, with the feasibility of incorporating genetic manipulations to modify the immunogenicity or reactogenicity of the EVs [68]. Together, these technological advances will benefit the development of bacterial membrane-coated nanoparticles with numerous implications toward effective and safe antibacterial vaccines.

## 4. Conclusions and Perspective

The cell membrane-coated nanoparticle platform translocates the entire cell exterior, including both lipids and membrane-associated proteins, from natural cells onto the surfaces of synthetic nanoparticles via a top-down approach. This platform technique integrates the rich functionalities of cellular membranes and the engineering controllability of synthetic nanomaterials into a hybrid, biomimetic nanoformulation that holds high promise for antibacterial vaccine development. When coated with RBC membranes, these biomimetic nanoparticles are able to sequester membrane-targeting protein toxins without denaturing the toxins, hence enabling structurally preserved toxins for immune processing. When coated with bacterial membrane, these nanoparticles mimic bacterial antigen presentation with tunable physicochemical properties and transport characteristics to elicit strong humoral and cellular immune responses against the source bacteria. These studies, together, have demonstrated the potential of cell membrane-coated nanoparticles as an emerging but highly effective antibacterial vaccine platform.

Since the initial discovery in 2011 [24], the cell membrane-coated nanoparticles have attracted much attention and are rapidly developed. On one hand, the membrane coating technology has been applied to functionalize nanoparticle cores made of an increasing number of materials, including gold [69,70], silica [71], and gelatin [50], which together provide a remarkable range of controllability useful for immune modulation. On the other hand, membranes from varying types of cells have also been collected as coating materials, unleashing extraordinary capability to harness natural functionalities for vaccine development besides anti-bacterial applications. In particular, polymeric nanoparticles coated with cancer cell membranes carry a full array of cancer cell membrane antigens with a high stability [72]. These nanoparticles enable colocalization and codelivery of multivalent tumor antigens with immunostimulatory adjuvants, resulting in an effective cancer vaccine that promotes tumor-specific immune responses.

Looking to the future, the cell membrane-coated nanoparticle system can be integrated with a series of other cutting-edge delivery technologies to further improve its use in vaccine development. For example, cell membrane-coated nanoparticles can be combined with delivery strategies already explored for noninvasive vaccine applications, such as microneedles [73] and hydrogel patches [74], for safe, rapid, and convenient vaccination. In addition, these nanoparticles can be integrated into biomaterial scaffolds that not only aid the recruitment of antigen presenting cells, but also create a nidus for prolonged immune activation [75,76]. In fact, RBC membrane-coated nanoparticles were recently integrated into hydrogels for local detoxification against bacterial infection, demonstrating the potential of such hybrid design for combined advantages of each building block [77]. Furthermore, a facile approach to synthesizing cell membrane-coated nanogels was recently reported by using cell membrane-derived vesicles to template inner core gelation [78]. This new approach uses cell membrane-derived vesicles to “guide” the core formation, hence effectively overcoming potential restriction imposed by the “coatability” of nanoparticle cores and thus significantly advancing the membrane coating technique. This would allow for readily encapsulation of a diverse range of cargos, such as immunostimulatory agents, into the cell membrane-coated nanoparticles in a fast and precisely controllable manner. Overall, cell membrane-coated nanoparticles as a new class of biomimetic nanoparticles are anticipated to open a range of unique opportunities toward novel vaccine development against bacterial infections.
